# Dietary diversity is associated with longitudinal changes in hippocampal volume among Japanese community dwellers

**DOI:** 10.1038/s41430-020-00734-z

**Published:** 2020-09-02

**Authors:** Rei Otsuka, Yukiko Nishita, Akinori Nakamura, Takashi Kato, Kaori Iwata, Chikako Tange, Makiko Tomida, Kaori Kinoshita, Takeshi Nakagawa, Fujiko Ando, Hiroshi Shimokata, Hidenori Arai

**Affiliations:** 1 Section of NILS-LSA, National Center for Geriatrics and Gerontology, Obu, Aichi Japan; 2 Department of Epidemiology of Aging, National Center for Geriatrics and Gerontology, Obu, Aichi Japan; 3 Department of Clinical and Experimental Neuroimaging, National Center for Geriatrics and Gerontology, Obu, Aichi Japan; 4 Japan Society for the Promotion of Science, Tokyo, Japan; 5 Faculty of Health and Medical Sciences, Aichi Shukutoku University, Nagakute, Aichi Japan; 6 Graduate School of Nutritional Sciences, Nagoya University of Arts and Sciences, Nisshin, Aichi Japan; 7 National Center for Geriatrics and Gerontology, Obu, Aichi Japan

**Keywords:** Risk factors, Epidemiology

## Abstract

**Background/Objectives:**

Dietary habits are known to affect health, including the rate of brain ageing and susceptibility to diseases. This study examines the longitudinal relationship between dietary diversity and hippocampal volume, which is a key structure of memory processing and is known to be impaired in dementia.

**Subjects/Methods:**

Subjects were aged 40–89 years (*n* = 1683, men: 50.6%) and participated in a 2-year follow-up study of the National Institute for Longevity Sciences-Longitudinal Study of Aging. Dietary intake was calculated from 3-day dietary records, and dietary diversity was determined using the Quantitative Index for Dietary Diversity at baseline. Longitudinal changes in hippocampal and total grey matter volumes were estimated by T1-weighted brain magnetic resonance imaging and FreeSurfer software. Estimated mean brain volume change in relation to dietary diversity score quintiles was assessed by the general linear model, adjusted for age, sex, education, smoking status, alcohol intake, physical activity, and comorbidities.

**Results:**

The mean (± standard deviation) % decreases in hippocampal and total grey matter volume during the 2-year follow-up were 1.00% (±2.27%) and 0.78% (±1.83%), respectively. Multivariate-adjusted decreases in total grey matter volume were associated with dietary diversity score (*p* = 0.065, *p* for trend = 0.017), and the % decrease in hippocampal volume was more strongly associated with the dietary diversity score: the estimated mean (± standard error) values were 1.31% (±0.12%), 1.07% (±0.12%), 0.98% (±0.12%), 0.81% (±0.12%), and 0.85% (±0.12%), according to dietary diversity quintiles in ascending order (*p* = 0.030, *p* for trend = 0.003).

**Conclusions:**

Among community dwellers, increased dietary diversity may be a new nutritional strategy to prevent hippocampal atrophy.

## Introduction

Dementia, including Alzheimer’s disease (AD), is a serious geriatric disease, and the number of dementia cases is increasing worldwide [1]. Since there are no curative treatments for dementia, more preventive strategies need to be established. Many lifestyle habits, including dietary factors, are thought to be associated with incident dementia. In fact, it was reported that the Mediterranean diet may help prevent dementia [2]. However, a recent systematic review reported that there was insufficient evidence from randomised controlled trials to draw conclusions regarding the beneficial effect of a Mediterranean diet on cognitive impairment or dementia [3].

The Japanese diet is characterised by a wide variety of seasonal ingredients and abundant aquaculture products [4]. An international comparative study reported that Japan demonstrated the highest values for healthy life expectancy and the second highest values for dietary diversity among 137 countries, and dietary diversity was positively associated with healthy life expectancy, even after controlling for gross domestic product [5]. We previously hypothesised that multiple nutritional deficiencies caused by an unbalanced diet might be a risk factor for cognitive decline and found that higher dietary diversity was associated with better cognitive function among older Japanese subjects [6]. However, we defined cognitive decline using the Mini-Mental State Examination [7]. A more objective measurement without the retest effect is needed to assess brain function.

In this study, we focused on structural neuroimaging analyses to explore brain regions associated with dietary factors. The hippocampus is a key structure in memory processing, the disturbance of which is a core symptom of dementia, and hippocampal atrophy occurs in AD and non-AD dementia disorders [7, 8]. Previous investigations have reported that greater adherence to the Mediterranean diet was associated with a larger hippocampal volume among 674 multiethnic older adults [9], a Western diet was associated with a smaller hippocampal volume after 4 years of follow-up among 255 older Australian subjects [10], and the long-term Healthy Eating Score was positively associated with hippocampal volume at the end of follow-up among 459 Whitehall II sub-study participants [11]. However, these were not large-scale studies (*n* < 1000), only one study measured brain volume change [10], and no study measured the effect of dietary diversity on hippocampal volume. This is the first study to examine the longitudinal relationship between dietary diversity and hippocampal volume to address the effects of daily diet on structural brain changes in a large sample of 1683 middle-aged and older Japanese community dwellers.

## Subjects and methods

Data for this survey were taken from the National Institute for Longevity Sciences-Longitudinal Study of Aging (NILS-LSA), which used detailed questionnaires, medical checkups, anthropometric measurements, physical fitness tests, and nutritional examinations to assess the normal ageing process over time. Participants in the NILS-LSA were randomly selected age- and sex-stratified individuals from a pool of community-dwelling residents in the National Center for Geriatrics and Gerontology neighbourhood areas of Obu City and Higashiura Town in Aichi Prefecture, Japan. The first wave of the NILS-LSA took place from November 1997 to April 2000 and included 2267 participants (1139 men, 1128 women; age range, 40–79 years). Subjects were followed up every 2 years and replaced by new, randomly recruited, age- and sex-matched participants when participants could not attend follow-up investigations, except for participants over 79 years old. Participants aged 40 years were also newly recruited every year. The Committee on the Ethics of Human Research of the National Center for Geriatrics and Gerontology approved the study protocol. Written informed consent was obtained from all participants. Details of the NILS-LSA were reported previously [12].

Participants in this study were selected from the sixth (July 2008 to July 2010) and seventh (July 2010 to July 2012) waves of the NILS-LSA study because 3-dimensional MRI data were available in these study waves. The following exclusion criteria were implemented among sixth-wave participants (*n* = 2302): (1) those who did not participate in the seventh wave (*n* = 315); (2) those who did not undergo MRI because of claustrophobia or other reasons or with defective MRI data at the sixth or seventh wave (*n* = 153); (3) those with a history of dementia at the sixth or seventh wave (*n* = 4); (4) those with a history of head surgery at the sixth or seventh wave (*n* = 21); (5) those whose MRI images showed apparent new cerebrovascular lesions diagnosed by a radiologist during the follow-up observation (*n* = 2); and (6) those with incomplete data on nutritional assessments (*n* = 113) or the lifestyle-related self-reported questionnaire (*n* = 11) at the sixth wave. Based on these criteria, longitudinal data from 1683 Japanese individuals (851 men, 832 women) aged 40–89 years were available for analysis.

### Nutritional assessments

After participation in the sixth wave of the study, subjects completed a 3-day dietary record for assessment of dietary intake, including the use of supplements. The dietary record was completed over 3 consecutive days (2 weekdays and 1 weekend day) [13]. Subjects completed the dietary record at home, and most returned it within 1 month. Food was weighed separately on a 1-kg kitchen scale (Sekisui Jushi, Tokyo, Japan) before being cooked, or portion sizes were estimated. Subjects used a disposable camera (27 shots; Fuji Film, Tokyo, Japan) to take photos of meals before and after eating. Dietitians used these photos to complete missing information in the dietary record. Information on any discrepancies and any required additional information were obtained via telephone calls to the subjects. Average food and nutrient intakes (including alcohol intake) over the 3 days were calculated according to the Standard Tables of Foods Composition in Japan 2010 and other sources [13, 14]. For supplements, almost 60% of subjects took supplements of some kind at the sixth wave [15], and we created an original supplement database based on the nutritional information of the supplement products used. However, the supplement database did not include phosphatidyl serine and/or choline, which have shown some benefits in terms of cognition [16, 17]. Furthermore, the supplement companies only published limited data on nutritional values. As a result, we did not consider the nutritional intake from supplements in this study.

Dietary diversity was determined using the Quantitative Index for Dietary Diversity (QUANTIDD) [18]. The QUANTIDD is calculated using the proportion of foods that contribute to total energy or the amount of foods and the number of food groups. The index ranges from 0 to 1, with lower scores indicating an unbalanced diet and higher scores indicating a more equal distribution of each food group. We calculated the score based on the amounts of 13 food groups (cereals, potatoes, beans, nuts and seeds, non-green-yellow vegetables, green-yellow vegetables, fruit, mushrooms, seaweed, fish and shellfish, meat, eggs, and milk and dairy products), excluding beverages and seasonings according to the Japanese food composition table [14].

### MRI data acquisition and processing

All MRI scans were performed with a 3.0-Tesla MRI scanner (Siemens Magnetom Tim Trio, Erlangen, Germany) with the Magnetisation-Prepared Rapid Gradient-Echo Imaging sequence [19]. High-resolution 3D T1-weighted images were acquired (TR/TE/TI = 1800/1.98/800 ms, 9-degree flip angle, 0.98 × 0.98 × 1.1 mm^3^ resolution, and 256 × 256 matrix). FreeSurfer version 5.3 (http://freesurfer.net) [20] was used for the cortical surface reconstructions and regional grey matter volume estimations. The technical details of these procedures have been described previously [7]. In short, the fully automated procedure in FreeSurfer involved preprocessing the subject’s image data, segmenting the cortical grey matter and white matter (GM/WM), tessellating the GM/WM junction, inflating the folded surface tessellation patterns, and automatically correcting the topologic defects. Subsequently, FreeSurfer parcellated the cerebral cortex into gyral based regions of interest according to the Desikan-Killiany atlas [21, 22] and performed automatic subcortical segmentation [23]. To reduce the confounding effect of inter-individual morphological variability, the longitudinal stream for FreeSurfer (Reuter et al. 2012) was used [24]. If any of the FreeSurfer processes, such as segmentation, failed, data for those cases were excluded from analyses. The volumes (cm^3^) of the left-right summation of the hippocampus and total grey matter were the dependent measures. To account for individual differences in head size and express units in cm^3^, individual regional grey matter volume, including hippocampal volume, was normalised by dividing it by the estimated total intracranial volume and then multiplying it by the mean estimated total intracranial volume of all subjects.

### Other measurements

Data on years of education, current smoking status, and chronic diseases (past and present hypertension, stroke, heart disease, diabetes mellitus, and dyslipidaemia) were collected by a self-administered questionnaire. The metabolic equivalent of task (MET) score, obtained from participant interviews conducted by trained interviewers using a semi-quantitative assessment, was used to assess 24-hour physical activity [25]. All measurements were assessed at baseline.

### Statistical analysis

Quintiles of the dietary diversity score were calculated by sex. Differences in proportions and continuous variables according to the quintiles of dietary diversity score were assessed using chi-squared analysis and analysis of variance, respectively. In post hoc analyses, trend tests of proportions and continuous variables according to quintiles of dietary diversity score were assessed using the Cochran–Armitage test or the general linear model, respectively.

Brain volume included hippocampal and total grey matter volume. Differences in volume at follow-up are expressed as two variables: difference over time (cm^3^) (volume at follow-up − volume at baseline) and percent decrease over time [(volume at baseline − volume at follow-up)/volume at baseline × 100].

Mean brain volume according to the quintiles of the dietary diversity score was estimated by the general linear model, adjusted for the following variables: (1) Model 1: sex, age (years, continuous), education (≤9, 10–12, or ≥13 years), current smoking status (yes or no), alcohol intake (mL/day, continuous), physical activity (METs *hr/day, continuous) and history of stroke, dyslipidaemia, diabetes, hypertension, and heart disease and (2) Model 2: Model 1 + baseline total grey matter volume or hippocampal volume.

Statistical analyses were conducted using Statistical Analysis System software version 9.3 (SAS Institute, Cary, NC, USA). *P* values < 0.05 and <0.1 were considered significant and marginally significant, respectively.

## Results

The mean (±SD) % decrease in hippocampal volume and total grey matter and the interval between the sixth and seventh waves were 1.00% (±2.27%), 0.78% (±1.83%), and 2.01 (±0.12) years, respectively. Therefore, the annual % decreases in hippocampal volume and total grey matter were 0.50% and 0.39%, respectively.

Table 1 shows baseline characteristics according to quintiles of dietary diversity score and indicates that higher dietary diversity was significantly associated with older age, lower physical activity, less smoking, and a significantly higher proportion of hypertension, dyslipidaemia, and diabetes. Regarding brain volume at baseline, subjects with higher dietary diversity had significantly lower hippocampal and total grey matter volume.

**Table 1:** Baseline characteristics according to quintiles of the dietary diversity score.

	Quintiles of dietary diversity score (*N* = 1683)						
	Q1 (low)	Q2	Q3	Q4	Q5 (high)	*P* value^a^	*P* for trend^b^
	(*N* = 336)	(*N* = 336)	(*N* = 337)	(*N* = 336)	(*N* = 338)		
Dietary diversity score (mean ± SD)	0.78 ± 0.07	0.85 ± 0.02	0.88 ± 0.01	0.90 ± 0.01	0.93 ± 0.01	<0.001	<0.001
Age (mean ± SD, years)	56.1 ± 12.2	58.2 ± 12.0	59.6 ± 12.0	62.4 ± 11.0	63.6 ± 11.0	<0.001	<0.001
Alcohol (mean ± SD, mL/day)	13.4 ± 21.9	13.6 ± 25.4	19.2 ± 31.2	12.8 ± 22.0	12.5 ± 22.5	0.002	0.544
Total physical activity (mean ± SD, METs*hr/day)	35.6 ± 4.0	35.0 ± 3.4	34.9 ± 3.5	34.7 ± 2.9	34.5 ± 3.2	0.001	<0.001
Men (*N*, %)	170,50.6%	170,50.6%	170,50.5%	170,50.6%	171,50.6%	0.999	–
Education (*N*, %)							
≤9 years	62,18.5%	62,18.5%	57,16.9%	42,12.5%	50,14.8%	0.368	–
10–12 years	133,39.6%	135,40.2%	123,36.5%	144,42.9%	135,39.9%		
≥13 years	141,42.0%	139,41.4%	157,46.6%	150,44.6%	153,45.3%		
Smoking status (*N*, %)							
Current	59,17.6%	49,14.6%	48,14.2%	36,10.7%	21,6.2%	0.0001	<0.001
Medical history (yes), (*N*, %)							
Stroke	10,3.0%	10,3.0%	9,2.7%	12,3.6%	15,4.4%	0.723	0.254
Heart disease	11,3.3%	7,2.1%	10,3.0%	13,3.9%	11,3.3%	0.754	0.559
Hypertension	65,19.4%	101,30.1%	107,31.8%	97,28.9%	99,29.3%	0.003	0.016
Dyslipidaemia	39,11.6%	69,20.5%	73,21.7%	76,22.6%	75,22.2%	0.001	0.001
Diabetes	12,3.6%	22,6.6%	20,5.9%	25,7.4%	38,11.2%	0.003	<0.001
Total grey matter volume^c^ (mean ± SD, cm^3^)	583.84 ± 34.14	578.24 ± 40.48	575.48 ± 35.79	571.12 ± 36.14	569.80 ± 39.21	<0.001	<0.001
Hippocampal volume^c^ (mean ± SD, cm^3^)	8.88 ± 1.02	8.78 ± 1.07	8.70 ± 0.99	8.72 ± 1.08	8.56 ± 1.13	0.003	<0.001

*SD* standard deviation.

^a^For continuous variables, analysis of variance was used; for categorical variables, χ^2^ test was used.

^b^In post hoc analyses, the general linear model was used for continuous variables, assigning dummy variables −2, −1, 0, 1, 2 to quintiles of dietary diversity score; Cochran–Armitage test was used for categorical variables.

^c^Crude (non-adjusted) value.

Table 2 shows baseline food and nutritional intake according to quintiles of the dietary diversity score. Subjects in the higher quintiles of dietary diversity scores ate significantly less cereal and more of the other 12 food groups (*p* for trend < 0.05). For nutrient intake, energy intake was not associated with the dietary diversity score, but subjects with dietary diversity scores in the higher quintiles ate significantly more protein and the other seven nutritional intake items, including sodium.

**Table 2:** Baseline food and nutritional intake according to quintiles of dietary diversity score.

	Quintiles of dietary diversity score (*N* = 1683)						
	Q1 (low)	Q2	Q3	Q4	Q5 (high)	*P* value^a^	*P* for trend^b^
	(*N* = 336)	(*N* = 336)	(*N* = 337)	(*N* = 336)	(*N* = 338)		
Food intake							
Cereals (g/day)	524.48 ± 156.97	475.38 ± 128.47	431.34 ± 111.38	405.40 ± 98.89	354.48 ± 96.96	<0.001	<0.001
Potatoes (g/day)	28.50 ± 30.50	37.93 ± 33.72	43.88 ± 35.30	48.92 ± 42.97	57.16 ± 41.76	<0.001	<0.001
Beans (g/day)	43.27 ± 54.87	60.98 ± 64.64	66.57 ± 53.72	65.44 ± 47.27	88.77 ± 57.11	<0.001	<0.001
Nuts and seeds (g/day)	2.20 ± 6.30	2.87 ± 7.61	3.04 ± 6.19	3.38 ± 5.08	4.26 ± 7.35	0.001	<0.001
Non-green-yellow vegetables (g/day)	157.26 ± 96.63	183.50 ± 95.84	205.56 ± 95.67	213.56 ± 91.61	218.72 ± 83.76	<0.001	<0.001
Green-yellow vegetables (g/day)	66.39 ± 51.57	95.59 ± 66.68	111.52 ± 76.71	123.41 ± 66.61	147.82 ± 70.08	<0.001	<0.001
Fruits (g/day)	69.09 ± 93.30	127.84 ± 133.12	139.77 ± 117.28	159.76 ± 101.19	179.60 ± 104.54	<0.001	<0.001
Mushrooms (g/day)	6.57 ± 8.44	10.66 ± 12.49	11.01 ± 12.64	13.05 ± 13.14	17.28 ± 18.52	<0.001	<0.001
Seaweed (g/day)	11.24 ± 15.38	13.09 ± 14.83	16.58 ± 17.27	19.22 ± 20.87	25.47 ± 23.38	<0.001	<0.001
Fish and shellfish (g/day)	68.07 ± 44.92	78.99 ± 48.08	82.18 ± 48.96	93.05 ± 49.22	106.51 ± 49.11	<0.001	<0.001
Meats (g/day)	66.88 ± 43.35	67.90 ± 44.67	70.19 ± 43.78	68.21 ± 38.14	75.42 ± 41.45	0.069	0.012
Eggs (g/day)	37.01 ± 25.57	38.87 ± 26.04	38.31 ± 22.23	42.46 ± 24.40	43.80 ± 24.51	0.001	<0.001
Milk and dairy products (g/day)	88.47 ± 132.88	123.34 ± 139.52	147.60 ± 122.46	168.72 ± 115.97	177.46 ± 99.32	<0.001	<0.001
Nutritional intake							
Energy (kcal/day)	1970.05 ± 433.53	2025.05 ± 409.90	2030.11 ± 365.83	2022.43 ± 373.69	2027.17 ± 414.47	0.250	0.084
Protein (g/day)	67.23 ± 14.62	72.55 ± 14.51	74.44 ± 13.84	77.85 ± 14.91	83.00 ± 16.22	<0.001	<0.001
Sodium (mg/day)	3953.19 ± 983.44	4039.25 ± 920.96	4176.29 ± 928.59	4302.93 ± 995.03	4422.71 ± 1043.12	<0.001	<0.001
Calcium (mg/day)	441.91 ± 206.91	529.70 ± 192.22	583.31 ± 172.69	631.68 ± 179.09	700.60 ± 189.84	<0.001	<0.001
Magnesium (mg/day)	234.69 ± 60.00	266.58 ± 65.76	281.80 ± 60.10	299.17 ± 65.64	321.63 ± 69.40	<0.001	<0.001
Iron (mg/day)	7.49 ± 2.09	8.39 ± 2.31	8.79 ± 2.08	9.42 ± 2.26	10.38 ± 2.50	<0.001	<0.001
Zinc (mg/day)	7.88 ± 2.02	8.38 ± 1.88	8.44 ± 1.82	8.75 ± 1.85	9.27 ± 2.19	<0.001	<0.001
Vitamin A (μg/day)	437.79 ± 357.23	594.60 ± 581.60	624.32 ± 637.34	722.64 ± 757.17	757.53 ± 608.48	<0.001	<0.001
Vitamin C (mg/day)	98.53 ± 81.83	117.14 ± 83.71	136.33 ± 111.15	136.09 ± 67.12	159.29 ± 84.20	<0.001	<0.001

Mean ± SD.

*SD* standard deviation.

^a^Analysis of variance was used.

^b^In post hoc analyses, the general linear model was used, assigning dummy variables −2, −1, 0, 1, 2 to quintiles of dietary diversity score.

Table 3 shows the multivariate-adjusted brain volume at baseline and the 2-year follow-up according to quintiles of the dietary diversity score. Subjects with higher dietary diversity had significantly lower hippocampal and total grey matter volumes (Table 1, crude analyses). However, additional age- and covariate-adjusted analyses indicated that baseline hippocampal and total grey matter volume were not significantly associated with baseline dietary diversity score. Differences in total grey matter between baseline and the 2-year follow-up had a significant association with the baseline dietary diversity score (model 1, *p* = 0.064, *p* for trend = 0.018). After additional adjustments were made for baseline total grey matter volume, these associations were attenuated (model 2, *p* = 0.102, *p* for trend = 0.028). In addition, the % decrease from baseline to follow-up also had a significant association with the baseline dietary diversity score (model 1, *p* = 0.065, *p* for trend = 0.017).

**Table 3:** Multivariate-adjusted^a^ brain volume at baseline and change at 2 years according to quintiles of baseline dietary diversity score.

		Quintiles of baseline dietary diversity score (*N* = 1683)						
		Q1 (low)	Q2	Q3	Q4	Q5 (high)	*P* value^b^	*P* for trend^b^
		(*N* = 336)	(*N* = 336)	(*N* = 337)	(*N* = 336)	(*N* = 338)		
Total grey matter volume								
Baseline (cm^3^)	Model 1	578.04 ± 1.76	575.54 ± 1.73	575.12 ± 1.73	574.72 ± 1.73	575.33 ± 1.74	0.698	0.265
Difference between two years^c^ (cm^3^)	Model 1	−5.92 ± 0.57	−4.76 ± 0.56	−3.73 ± 0.56	−4.24 ± 0.56	−4.03 ± 0.57	0.064	0.018
	Model 2	−5.79 ± 0.56	−4.77 ± 0.56	−3.77 ± 0.55	−4.30 ± 0.56	−4.05 ± 0.56	0.102	0.028
Decrease % from baseline to follow-up period (two years)^d^ (%)	Model 1	1.02 ± 0.10	0.83 ± 0.10	0.64 ± 0.10	0.74 ± 0.10	0.69 ± 0.10	0.065	0.017
Hippocampal volume								
Baseline (cm^3^)	Model 1	8.702 ± 0.049	8.700 ± 0.048	8.685 ± 0.048	8.827 ± 0.048	8.732 ± 0.048	0.233	0.333
Difference between two years^c^ (cm^3^)	Model 1	−0.109 ± 0.010	−0.088 ± 0.010	−0.082 ± 0.010	−0.070 ± 0.010	−0.072 ± 0.010	0.042	0.004
	Model 2	−0.109 ± 0.010	−0.089 ± 0.010	−0.083 ± 0.010	−0.068 ± 0.010	−0.072 ± 0.010	0.029	0.003
Decrease % from baseline to follow-up period (two years)^d^ (%)	Model 1	1.31 ± 0.12	1.07 ± 0.12	0.98 ± 0.12	0.81 ± 0.12	0.85 ± 0.12	0.030	0.003

Mean ± SE.

*SE* standard error.

^a^Adjusted variables.

^b^In post hoc analyses, the general linear model was used, assigning dummy variables −2, −1, 0, 1, 2 to quintiles of dietary diversity score.

^c^The difference (cm^3^) was calculated from this formula: “Volume at follow-up” - “Volume at baseline”.

^d^Decrease % from baseline to follow-up period (two years) was calculated from this formula: (“Volume at baseline” − “Volume at follow-up”)/“Volume at baseline” *100.

Model 1: baseline age, sex, education, smoking status, alcohol intake, physical activity and history of stroke, dyslipidaemia, diabetes, hypertension, and heart disease.

Model 2: Model 1 + baseline total grey matter volume or hippocampal volume.

Regarding hippocampal volume, the estimated mean (±standard error) volume difference between 2 years was significantly associated with the baseline dietary diversity score: −0.109 cm^3^ (±0.010), −0.088 cm^3^ (±0.010), −0.082 cm^3^ (±0.010), −0.070 cm^3^ (±0.010), and −0.072 cm^3^ (±0.010), according to the dietary diversity quintiles in ascending order (model 1, *p* = 0.042, trend *p* = 0.004). Even after adjusting for baseline hippocampal volume, the negative association was maintained (model 2). The percent decrease from baseline to the follow-up period was also significantly associated with the baseline dietary diversity score: 1.31% (±0.12%), 1.07% (±0.12%), 0.98% (±0.12%), 0.81% (±0.12%), and 0.85% (±0.12%), according to the dietary diversity quintiles in ascending order (model 1, *p* = 0.030, trend *p* = 0.003).

## Discussion

This is the first large-scale longitudinal study focused on dietary diversity and brain morphology and showed that higher dietary diversity was associated with a smaller decrease in hippocampal volume among community-dwelling Japanese adults.

Regarding the normal ageing process, meta-analyses of longitudinal hippocampal atrophy reported a mean annual atrophy rate of 0.85% (95% confidential interval: 0.63–1.07) [26] and a mean annual grey matter volume change of 0.424% in men and 0.298% in women among community-dwelling Japanese [27]. In our cohort, the mean annual % decreases in hippocampal volume and total grey matter were almost 0.50% and 0.39%, respectively, which are consistent with the meta-analysis; therefore, our cohort is representative of Japanese people.

The multivariate-adjusted % decrease in hippocampal volume (2 years) ranged from 0.81 in the fourth quintile to 1.31 in the first quintile of dietary diversity, and the degree was attenuated by the baseline dietary diversity level. In other words, hippocampal atrophy progressed in all quintile groups of the dietary diversity score, but atrophy might have been attenuated by eating a variety of foods.

In our cohort study, the dietary diversity score was positively associated with vegetables, fruits, fish and dairy products and negatively associated with cereals. Previous epidemiologic studies also reported that greater adherence to a Mediterranean diet rich in vegetables, legumes, cereals, fish, and fruits [9], and healthy eating patterns including vegetables, fruit, whole grains, nuts, and legumes [11] were positively associated with hippocampal volumes in Western subjects, and a diet rich in roasted meat, sausages, hamburgers, and soft drinks was negatively associated with hippocampal volumes in Western subjects. We could not examine the association between brain atrophy and the traditional Mediterranean Diet Score based on daily consumption of seasonal fruit and vegetables, olive oil, cheese, fish, eggs, nuts, wine, and some chicken [28], as Japanese individuals consume less olive oil, cheese, nuts, and wine, which are the main components of the Mediterranean diet. Most previous results regarding the positive association of diets rich in vegetables, fruits, and legumes with hippocampal volume were similar to the findings of our study, but dietary patterns were different. Dietary diversity used in this study was calculated based on the proportion of 13 food groups. However, we did not define favourable or unfavourable foods [29] because our hypothesis was that multiple nutritional deficiencies caused by an unbalanced diet might be a risk factor for hippocampal atrophy. Hippocampal atrophy occurs during the progression of AD [30], and higher dietary diversity might be an effective nutritional strategy for preventing dementia.

Two main dietary mechanisms contributing to brain ageing should be considered. One involves metabolic mechanisms; for example, subjects with higher dietary diversity eat more protein, fat, minerals, and vitamins. These nutrients have anti-inflammatory and antioxidant effects and have been shown to affect neurogenesis of the hippocampus in humans [31, 32]. The second mechanism is dietary behaviour, as eating a variety of foods requires health consciousness and healthy activities such as preparing meals, which includes shopping, cooking, and planning a menu at least 3 times per day, two intentional and complex instrumental activities that require multiple cognitive processes [6]. Therefore, it is possible that eating or preparing a variety of foods may have a favourable effect on brain ageing.

In this study, a multivariate-adjusted % decrease in hippocampal volume, in comparison to that in total grey matter volume, was more strongly associated with the baseline dietary diversity score. The precise mechanism of these findings is unknown, but differences may depend on differences in the degree of brain atrophy. Previous studies have shown annual hippocampal and grey matter volume changes of 0.85% and 0.298–0.424%, respectively, in the context of normal ageing [26, 27]. The degree of brain atrophy was higher in the hippocampus than in total grey matter, similar to our results, which may indicate that hippocampal atrophy is more accelerated with ageing, and statistical changes may be more detectable.

The present study has several strengths. First, our cohort study was larger (almost 1700 individuals) than previous studies, and this study first demonstrated longitudinal brain atrophy and its association with daily diet diversity among Asians. We assessed dietary diversity based on the proportion of 13 food groups and showed that higher dietary diversity was positively associated with recommended nutrient intake [29]. It is possible that dietary patterns, such as eating several kinds of foods or more side dishes, can help prevent brain atrophy. These changes could represent easy and effective public nutritional strategies for community dwellers. Even in community dwellers who may not have access to professionals with nutritional knowledge or subjects who cannot prepare meals containing a variety of foods, advising them to eat a greater variety of foods represents simple advice that might be easy to put into practice.

Several limitations of the present study must also be considered. Food intake is easily changeable and affected by various factors associated with ageing. In fact, our previous study conducted in the same cohort showed that dietary diversity increased in both men and women until middle age (55 years in men, 44 years in women) and declined in women aged 63–79 years [33]. However, we did not evaluate the cumulative effects of previous dietary patterns or diversity in this study. Second, the Japanese intake of meat and dairy products is considered low by global standards [5]. In addition, Japanese individuals eat a wider diversity of foods [5]. Therefore, the present findings may not be generalised to Western populations who consume larger amounts of meat and a smaller variety of foods.

This 2-year prospective cohort study shows that higher dietary diversity is negatively associated with hippocampal atrophy. The mean % decrease in hippocampal volume during the 2 years was 1.00%, and the difference in the dietary diversity score between the fourth and first quintile was 0.5% (1.31 − 0.81%), which represents a large change; therefore, eating a variety of foods could attenuate hippocampal atrophy. In sub-analyses, we examined the longitudinal association between the baseline dietary diversity score and the 2-year change in information processing speed using the digit symbol substitution test [34], and there was no statistically significant association between these variables (data not shown). It is possible that a 2-year longitudinal study might be insufficient not only to evaluate morphological changes but also to detect cognitive decline. More longitudinal studies and studies including non-Asians are needed to confirm any association between dietary diversity and brain atrophy and cognitive function.

In conclusion, eating a variety of foods may be a new effective nutritional strategy to prevent hippocampal atrophy among community dwellers.
